# Predictive Value of Two Polymorphisms of ERCC2, rs13181 and rs1799793, in Clinical Outcomes of Chemotherapy in Gastric Cancer Patients: A Meta-Analysis

**DOI:** 10.1155/2018/3947626

**Published:** 2018-11-19

**Authors:** Mengxi Li, Yan Zhao, Erjiang Zhao, Kunlun Wang, Weiquan Lu, Ling Yuan

**Affiliations:** Affiliated Tumor Hospital of Zhengzhou University, Henan Tumor Hospital, Zhengzhou, Henan 450008, China

## Abstract

**Background:**

Several researchers have investigated the relationship between ERCC2 rs13181 and rs1799793 polymorphisms and chemotherapy efficacy in terms of tumour response and prognosis in gastric patients. However, the published data have shown inconsistencies.

**Methods:**

PubMed, Elsevier, and Chinese National Knowledge Infrastructure databases were searched for relevant articles published before August 1, 2017. Thirteen studies including 3096 gastric cancer patients treated with chemotherapy were included.

**Results:**

For rs1799793, in the overall analyses, no relationships were found between four genetic models and clinical response (AA vs. GG: OR = 1.17, 95% CI, 0.70–1.95; GA vs. GG: OR = 0.94, 95% CI, 0.69–1.27; GA + AA vs. GG: OR = 1.12, 95% CI, 0.85–1.46; and AA vs. GG + GA: OR = 1.24, 95% CI, 0.81–1.92). In stratified analyses, the results remained negative. We also found no relationship between each of the genetic models and overall survival time in the overall analyses. In the stratified analyses, for Asians, the A carrier genotype might be more closely associated with shorter survival time and higher risk of death for patients than the GG genotype (AA vs. GG: HR = 1.77, 95% CI, 1.20–2.6; GA + AA vs. GG: HR = 1.62, 95% CI, 1.26–2.09), but the results were negative for Caucasians. No significant relationships were found between the rs13181 polymorphism and OR or OS.

**Conclusions:**

This meta-analysis suggested that the ERCC2 rs1799793 polymorphism might be a predictor of prognosis in gastric cancer patients subjected to platinum-based chemotherapy.

## 1. Introduction

Gastric cancer is a serious public health problem worldwide, and its morbidity and mortality rates rank fourth and second, respectively, among all tumours [1]. Currently, surgery is the primary management modality for patients with early stage and locally advanced gastric cancer, but most patients with gastric cancer either are diagnosed at an advanced stage or develop a relapse after curative surgery [2]. Apart from supportive care and palliative radiotherapy for patients with advanced and localized metastasis, systemic chemotherapy is the only treatment option available [3]. Platinum (in the form of oxaliplatin, cisplatin, etc.) combined with fluoropyrimidines (5-fluorouracil, capecitabine, S-1, etc.) has been most commonly used in chemotherapy regimens for patients with gastric cancer so far [4–7]. However, the treatment response and prognosis in response to chemotherapy vary remarkably among individual patients. Some genetic factors have been speculated to affect the clinical outcomes of patients; one example of such a genetic factor is excision repair cross-complementing group 2 (ERCC2).

ERCC2, encoded by a gene located at chromosome 19q13.3, is an ATP-dependent helicase that mediates DNA unwinding for the initiation of nucleotide excision repair (NER) [8]. The NER pathway, one of the well-known DNA repair pathways, maintains genomic integrity by removing bulky DNA lesions or interstrand adducts induced by exogenous and/or endogenous factors [9]. The DNA repair system plays a crucial role in maintaining stable cellular functions and genomic integrity by reversing the DNA damage induced by various endogenous and/or exogenous factors including therapeutic agents; therefore, the host DNA repair capacity may contribute to the outcomes of cancer patients [10, 11]. For these reasons, whether there are connections between ERCC2 and the clinical outcomes of gastric cancer patients in response to platinum-based chemotherapy is a hot question.

In recent years, many researchers have investigated possible relationships between two ERCC2 polymorphisms, rs13181 and rs1799793, and treatment response and prognosis, which are indicators of chemotherapy efficacy, in patients with gastric cancer [12–24]; however, most of these studies have been inconclusive. Therefore, this meta-analysis was conducted to evaluate the abovementioned relationships.

## 2. Materials and Methods

### 2.1. Search Strategy

We conducted a comprehensive literature search of PubMed, Elsevier, and Chinese National Knowledge Infrastructure (CNKI) databases from inception until August 1, 2017, using the following terms: (“gastric cancer” or “stomach cancer” or “gastric carcinoma” or “stomach carcinoma” or “gastric Neoplasm” or “stomach Neoplasm”) and (“excision repair cross-complementing group 2” or “ERCC2” or “xeroderma pigmentosum group D” or “XPD”) and (“chemotherapy”). No restrictions on publication date or language were imposed. Furthermore, the bibliographies of the relevant reviews and articles were reviewed manually to identify additional eligible studies. The current study was conducted according to the PRISMA guidelines for systematic reviews and meta-analyses [25].

### 2.2. Inclusion and Exclusion Criteria

Studies that fulfilled all three of the following inclusion criteria were considered eligible: (1) the gastric cancer patients were treated with chemotherapy alone; (2) ERCC2 rs13181 or rs1799793 polymorphism was genotyped; and (3) the studies provided sufficient data of clinical outcomes (ORR, OS, and HR with corresponding to 95% CIs). The exclusion criteria were as follows: (1) repeated publications; (2) studies comprising reviews or meta-analyses; (3) obviously irrelevant studies; (4) studies not relevant to ERCC2; (5) studies including patients not treated with chemotherapy alone; and (6) studies with insufficient data.

### 2.3. Quality Assessment

Two investigators (Mengxi Li and Yan Zhao) assessed the quality of each study using the Newcastle-Ottawa Quality Assessment Scale in order to control the quality of this meta-analysis (Table 1). This scale provides scores according to patient selection, study comparability, follow-up, and outcome. NOS scores of 1–3, 4–6, and 7–9 were defined as low-, intermediate-, and high-quality studies, respectively. Any discrepancies were resolved by consensus.

### 2.4. Data Extraction

Two investigators (Li and Zhao) carried out the screening and extracted relevant data from each of the eligible studies independently. Discrepancies were resolved by consultation with a third investigator. For each study, the following data were collected: the first author's name, publication year, country, ethnicity of the study participants (Asian and Caucasian), number of patients, age, TNM stage, evaluation criterion (WHO and RECIST), outcomes (ORR, OS, and HR with corresponding 95% CIs), and the number of responders and nonresponders with different genotypes.

### 2.5. Statistical Analysis

Four genetic models were analysed for each gene site in this meta-analysis. For Lys751Gln (rs13181, A > C): we analysed homozygote genetic models (CC vs. AA), performed heterozygote comparison (AC vs. AA), and studied a dominant model (AC + CC vs. AA) and a recessive model (CC vs. AA+AC); for Asp312Asn (rs1799793, G > A): we analysed homozygote genetic models (AA vs. GG), performed heterozygote comparison (GA vs. GG), and studied a dominant model (GA + AA vs. GG) and a recessive model (AA vs. GG + GA). To evaluate the strength of the association between the ERCC2 rs13181 and rs1799793 polymorphisms and the rate of response to chemotherapy, the gastric cancer patients were classified into responders (complete response (CR) + partial response (PR)) and nonresponders (progressive disease (PD) + stable disease (SD)) according to RECIST [26] or WHO criteria [27]. The crude odds ratios (ORs) with 95% CIs were computed and compared between the responders and the nonresponders. For OS, the hazard risks (HRs) and CIs extracted from the raw data of the included articles were calculated to estimate the pooled HRs and 95% CIs in the homozygote genetic model, heterozygote comparison, and dominant comparison. The Chi-square-based Q-test and *I*^2^ statistics were used to estimate between-study heterogeneity. If the *I*^2^ index > 50% for the Q-test, the random effects model (DerSimonian-Laird method) was applied to estimate the pooled OR; else, the fixed-effects model (Mantel-Haenszel method) was used [28]. In addition, subgroup analyses were performed based on ethnicities (Caucasians and Asians). The potential publication bias of the literature was evaluated using funnel plots and Egger's linear regression method [29]. All statistical analyses were performed using the STATA software (version 12.0; STATA Corporation, College Station, TX, USA) with two-sided *P* values.

## 3. Results

### 3.1. Characteristics of the Studies

**Figure 1:**
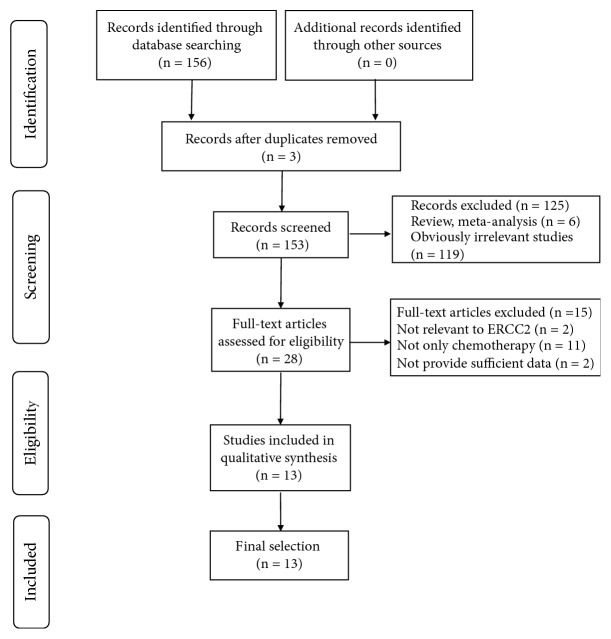
The flow chart of included studies in this meta-analysis.

The primary search strategy yielded 156 potentially relevant publications. After excluding duplicated papers, reviews, meta-analyses, and irrelevant studies, the remaining 28 articles were assessed further. These articles underwent full-text review; two articles were excluded as they were not relevant to ERCC2; 11 were excluded as the patients were subjected to additional treatments other than chemotherapy, and two were excluded because of insufficient data. Finally, 13 articles including 3096 gastric cancer patients met the inclusion criteria and thus were included in this meta-analysis (Figure 1). Of them (Table 2), two studies were conducted on Caucasian patients and 11 on Asians. The sample sizes ranged from 73 to 415. Eleven studies reported ORR and ten reported the OS and HR.

### 3.2. ORR of ERCC2 rs13181 and rs1799793 Polymorphisms

Eleven studies (Table 2) including 2898 gastric cancer patients reported an association between ERCC2 rs13181 and rs1799793 polymorphisms and the clinical response to platinum-based chemotherapy.

**Figure 2:**
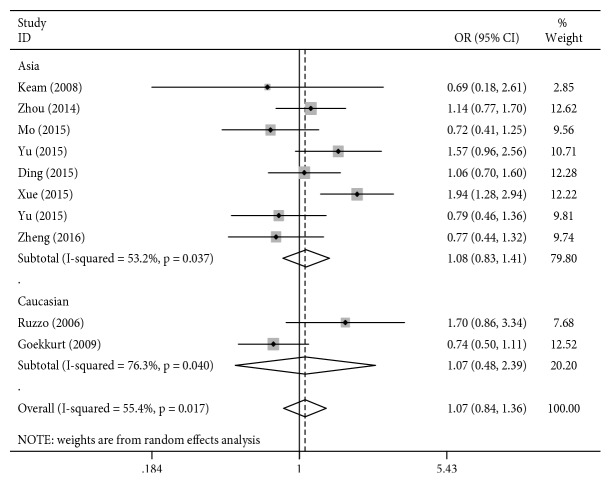
Forest plot for association of the ERCC2 rs13181 polymorphism with the treatment response to chemotherapy in gastric cancer patients (AC + CC VS. AA).

For rs13181, the results of the meta-analysis are presented in Table 3 and Figure 2. In the overall analysis, no significant associations were observed between the ERCC2 rs13181 polymorphism and clinical response to platinum-based chemotherapy for any of the genetic models (*P* > 0.05, Table 3). Subgroup analysis by ethnicity also revealed a negative correlation with response to platinum-based chemotherapy in the Asian and Caucasian gastric cancer patients (*P* > 0.05, Table 3).

**Figure 3:**
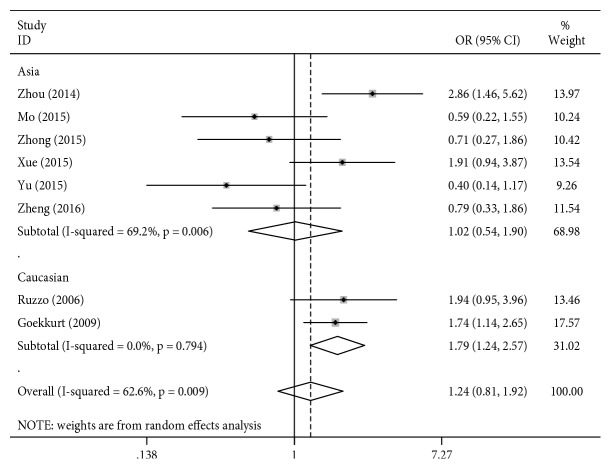
Forest plot for association of the ERCC2 rs1799793 polymorphism with the overall survival to chemotherapy in gastric cancer patients (AA VS. GG + GA).

The results from the meta-analysis for rs1799793 are presented in Table 4 and Figure 3. In the overall analysis, no significant associations were observed between the ERCC2 rs1799793 polymorphism and clinical response to platinum-based chemotherapy for any of the genetic models (*P* > 0.05, Table 4). However, subgroup analysis by ethnicity revealed a positive correlation with clinical response to platinum-based chemotherapy in the Caucasian gastric cancer patients (for AA vs. GG + GA: OR = 1.79, 95% CI = 1.24–2.57, *P* = 0.002, Figure 3; all the other *P* values > 0.05, Table 4), but the results showed no associations between the ERCC2 rs1799793 polymorphisms and ORR in Asian patients (*P* > 0.05, Table 4).

### 3.3. OS of ERCC2 rs13181 and rs1799793 Polymorphisms

Ten studies (Table 5) including 2502 gastric cancer patients revealed an association between the ERCC2 rs13181 polymorphism and OS. The results of the meta-analysis showed no association between the ERCC2 rs13181 polymorphism and OS for three genetic models in the overall analysis. The overall analysis amounts to ethnicity-based subgroup analysis in this case because all the patients included in the studies involving OS were Asian (*P* > 0.05, Table 3).

**Figure 4:**
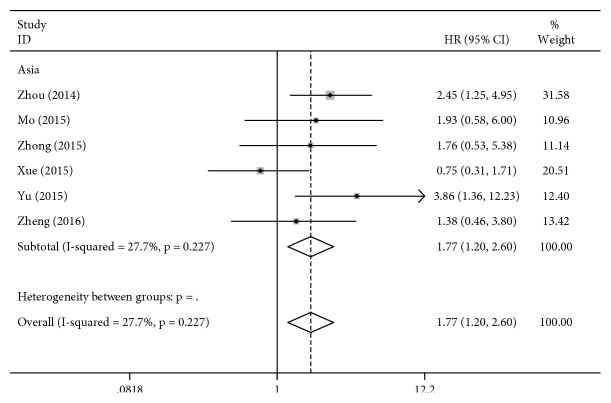
Forest plot for association of the ERCC2 rs1799793 polymorphism with the overall survival to chemotherapy in gastric cancer patients (AA VS. GG).

**Figure 5:**
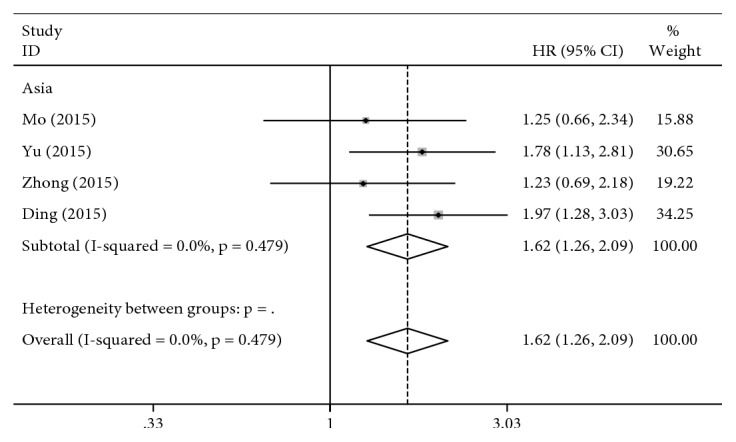
Forest plot for association of the ERCC2 rs1799793 polymorphism with the overall survival to chemotherapy in gastric cancer patients (GA + AA VS. GG).

Ten studies (Table 5) including 2675 gastric cancer patients were identified for the association between the ERCC2 rs1799793 polymorphism and OS. The results of the meta-analysis indicated that the ERCC2 rs1799793 polymorphism was associated with OS for three genetic models in the overall analysis (Table 4). The overall analysis amounts to ethnicity-based subgroup analysis in this case because all the patients included in the studies showing OS were Asian (for AA vs. GG: HR = 1.77, 95% CI = 1.20–2.60, *P* = 0.004, Figure 4; for GA + AA vs. GG: HR = 1.62, 95% CI = 1.26–2.09, *P* < 0.001, Figure 5; other *P* values > 0.05).

### 3.4. Publication Bias

**Figure 6:**
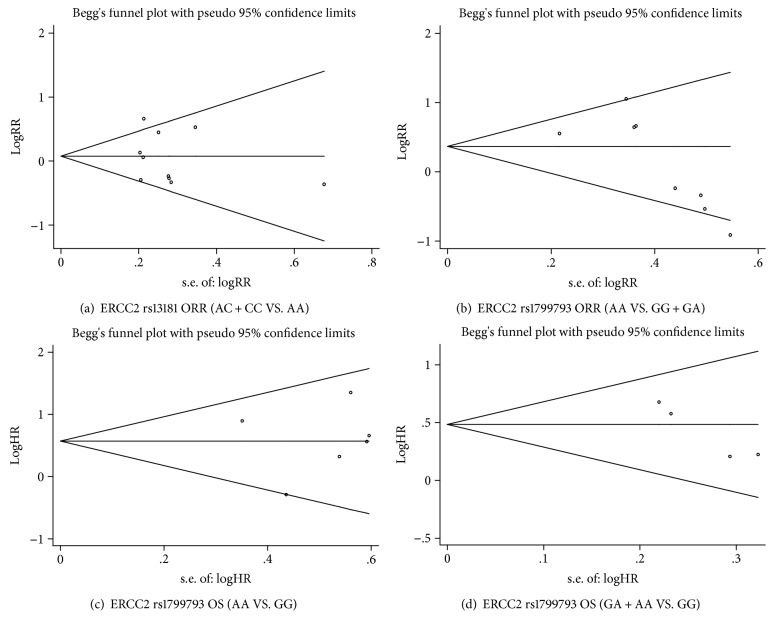
Begg's funnel plot for publication bias analysis.

The potential publication bias of the literature was evaluated using funnel plot analysis and Egger's test. The shapes of the funnel plots were approximately symmetrical. Egger's test revealed almost no publication bias for any of the genetic models, except for the GA and AA genotypes in comparison with the GG genotype of rs1799793 for OS (Tables 3 and 4; Figure 6).

### 3.5. Sensitivity Analysis and Metaregression

**Figure 7:**
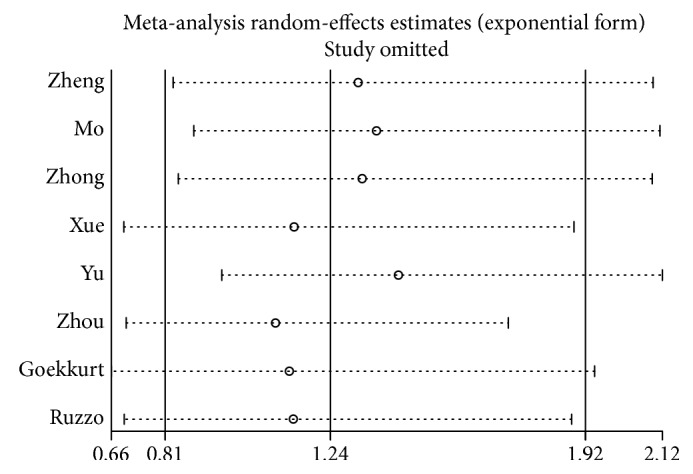
Sensitivity analysis of ERCC2 rs1799793 ORR (AA VS. GG + GA).

**Figure 8:**
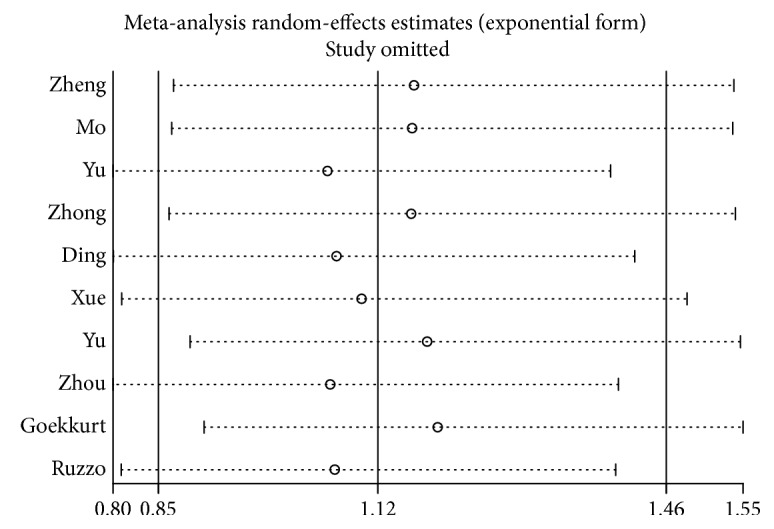
Sensitivity analysis of ERCC2 rs1799793 ORR (GA + AA VS. GG).

**Figure 9:**
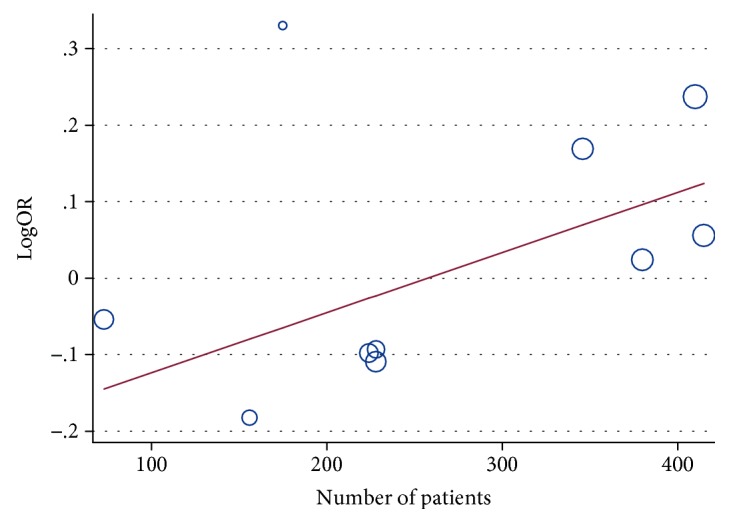
Metaregression of ERCC2 rs13181 ORR (AC + CC VS. AA).

**Figure 10:**
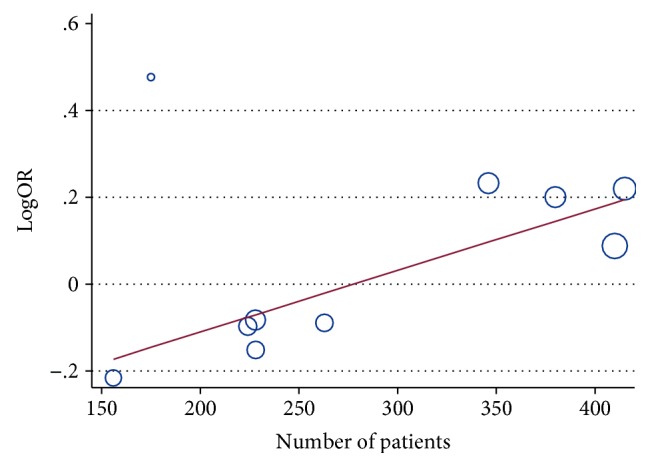
Metaregression of ERCC2 rs1799793 ORR (GA + AA VS. GG).

We conduct a sensitivity analysis to evaluate whether the differences between studies induced instability in the meta-analysis or not. The results suggested that the meta-analysis was stable (Figures 7 and 8). We also performed a metaregression to assess potential heterogeneity of individual study; the results showed that the number of cases in each articles may be the source of potential heterogeneity (Figures 9 and 10).

## 4. Discussion

Platinum-based chemotherapy agents represent the most active anticancer agents in clinical use, both in individual or in combination therapies with reasonable success, inducing DNA adducts. These platinum-DNA adducts cause distortion of the DNA double helix, activate a cellular DNA damage response, and lead to tumour cell death. ERCC2 is an important member of the NER pathway. It can remove platinum-DNA adducts with platinum-based chemotherapy. Polymorphisms in ERCC2 are closely related to the efficacy of chemotherapy drugs in gastric cancer patients.

Several researchers have reported a relationship between ERCC2 rs13181 and rs1799793 polymorphisms and chemotherapy efficacy in gastric cancer patients [12–24]. In 2014, a study by Yu et al. revealed that the AA genotype of the ERCC2 rs1799793 polymorphism is associated with better response to chemotherapy in gastric cancer patients [19]. Many other related findings were also reported in 2015. Zhong et al. found that the GA + AA genotypes of ERCC2 rs1799793 are associated with a significantly better response to chemotherapy compared with the GG genotype and that the GA + AA genotypes are significantly associated with a lower risk of mortality from gastric cancer compared with the GG genotype [15]. Ding et al. also showed that gastric cancer patients with the ERCC2 rs1799793 GA genotype tended to have shorter OS than those with the GG genotype [16]. However, Xue et al. found that the ERCC2 rs13181 and rs1799793 polymorphisms are not correlated with response to FOLFOX chemotherapy but revealed a significantly increased risk of death from gastric cancer among patients with the ERCC2 rs1799793 AA genotype compared with patients with the GG genotype in terms of overall survival [18]. Another study by Mo et al. found that patients with the ERCC2 rs1799793 GA + AA genotype exhibited longer survival times than did those with the GG genotype [13]. However, the meta-analysis by Zhang et al. found no statistically significant difference in the effective clinical response and overall survival between any of the genetic models of Lys751Gln (rs13181) [30]. In contrast, the meta-analysis conducted by Yin et al. showed that for ERCC2 rs13181 T > G, the G allele was associated with reduced objective response in all the patients, in all subgroups of Caucasians. For OS, the significance was observed only in Caucasian subgroups [31]. This study included patients with gastric and colorectal cancer, but the results for these two types of patients were not analysed separately. It is possible that the reason for the inconsistency between our findings and the results of this study was the inclusion of colorectal cancer patients. These studies by Zhang et al. and Yin et al. contained small sample sizes, and the patients were not treated with chemotherapy alone. In addition, neither of the two studies were focused on the relationship between ERCC2 rs1799793 and the clinical response of gastric cancer patients to platinum-based chemotherapy.

Thus, with a series of new studies published, we conducted a meta-analysis to derive a more precise and comprehensive assessment of the relationship of ERCC2 rs13181 and rs1799793 polymorphisms with the efficacy and clinical outcomes of gastric cancer patients treated with platinum-based chemotherapy. This is the first meta-analysis evaluating not only ERCC2 rs13181 but also rs1799793. Thirteen studies including 3096 gastric cancer patients identified the association between ERCC2 rs13181 or rs1799793 polymorphisms and clinical response to platinum-based chemotherapy drugs. All the patients included in our article were treated with chemotherapy alone, without surgery or radiotherapy. Our meta-analysis revealed some correlations between the recessive model of rs1799793 and response to platinum-based chemotherapy in Caucasian gastric cancer patients (AA vs. GG + GA: OR = 1.79, 95% CI 1.24–2.57). However, these results were not observed in patients with the other three genotypes. Therefore, the above data need to be handled with caution. Additionally, the A allele carrier of rs1799793 might be more closely associated with shorter survival time and higher risk of death than the GG genotype in Asian gastric cancer patients (AA vs. GG: HR = 1.77, 95% CI = 1.20–2.60; GA + AA vs. GG: HR = 1.62, 95% CI = 1.26–2.09) but not in Caucasian patients. This phenomenon may be attributable to the fact that the ERCC2 rs1799793 polymorphism causes an amino acid substitution from aspartic acid (asp) to asparagine (asn), which leads to increased activity of synthesis enzymes and increases their ability to detoxify and excrete platinum-based agents. This reduces the concentration of platinum-based agents in tumour cells, thereby decreasing the sensitivity of the cells. Thus, ERCC2 rs1799793 may be a useful biomarker in predicting the clinical outcomes of gastric cancer patients in response to platinum-based chemotherapy.

There are several limitations to our meta-analysis. Firstly, because of the differences in several characteristics of the design, histological type, tumour stage, gender, age, and follow-up time of the patients included in the studies, our results should be carefully evaluated. Secondly, in the subgroup analysis by ethnicity, the sample size was small for Caucasian patients, which may lead to insufficient power to assess the true correlation. Therefore, the results of the current meta-analysis should be applied with caution in Caucasian patients. Moreover, the ethnicity factor should be considered if a specific platinum-based chemotherapeutic regimen for gastric cancer patients is to be used in the future. Thirdly, because the patients included in the studies had received chemotherapy alone, without radiotherapy or surgical excision, the number of articles that could be included in our meta-analysis was greatly reduced, which may have led to some errors.

Nevertheless, this meta-analysis suggests that the ERCC2 rs1799793 polymorphism is a predictor of prognosis in Asian gastric cancer patients undergoing platinum-based chemotherapy. Furthermore, gastric cancer patients with the A allele (AA and GA) may be more likely to show shorter survival time and higher risk of death than those with GG genotypes. However, the use of the ERCC2 rs1799793 polymorphism as a predictive factor of prognosis in personalized chemotherapy treatment requires further verification from large well-designed pharmacogenetics studies.

## 5. Conclusion

This meta-analysis suggested that the ERCC2 rs1799793 polymorphism might be a predictor of prognosis in gastric cancer patients subjected to platinum-based chemotherapy.

## Figures and Tables

**Table 1:** Newcastle-Ottawa quality assessment scale.

Selection
(1) Representativeness of the exposed cohort
(a) Truly representative of the average “GC patient” in the community (1 star)
(b) Somewhat representative of the average “GC patient” in the community (1 star)
(c) Selected group of users (e.g., nurses, volunteers)
(d) No description of the derivation of the cohort
(2) Selection of the nonexposed cohort
(a) Drawn from the same community as the exposed cohort (1 star)
(b) Drawn from a different source
(c) No description of the derivation of the nonexposed cohort
(3) Ascertainment of exposure (proof of GC and platinum-based chemotherapy)
(a) Secure record (e.g., chemotherapy records) (1 star)
(b) Structured interview
(c) Written self-report
(d) No description
(4) Demonstration that outcome of interest was not present at start of study
(a) Yes (1 star)
(b) No
Comparability
(1) Comparability of cohorts on the basis of the design or analysis
(a) Study controls for “chemotherapy regimens” (1 star)
(b) Study controls for any additional factor (age, stage, etc.) (1 star)
Outcome
(1) Assessment of outcome (death or recurrence)
(a) Independent blind assessment (1 star)
(b) Record linkage (1 star)
(c) Self-report
(d) No description
(2) Was follow-up long enough for outcomes to occur? (death or recurrence)
(a) Yes (sufficient follow-up time was selected to observe the outcome) (1 star)
(b) No
(3) Adequacy of follow-up of cohorts
(a) Complete follow-up all subjects accounted for (1 star)
(b) Subjection lost to follow-up unlikely to introduce bias-small number lost “25%” or description provided of those lost (1 star)
(c) Follow-up rate “75%” and no description of those lost
(d) No statement

GC: gastric cancer.

**Table 2:** Characteristics of eligible studies included in this meta-analysis.

Number	Study	Year	Country	Ethnicity	Number of patients	Age	Cancer types	Chemotherapeutic	TNM stage	Evaluation criterion	Outcomes	ERCC2 rs13181				ERCC2 rs1799793			
												AA	AC	CC	AC + CC	GG	GA	AA	GA + AA
1	Zheng	2016	China	Asia	224	57.4 ± 9.20	Gastric cancer	Platinum-based chemotherapy	I-IV	WHO	ORR	77/39^a^	51/33^a^	14/10^a^	_	82/42^a^	46/30^a^	14/10^a^	_
2	Mo	2015	China	Asia	228	56.65 ± 11.52	Gastric cancer	Platinum-based chemotherapy oxaliplatin	I-IV	RECIST	ORR	82/34^a^	54/29^a^	17/12^a^	71/41^a^	87/38^a^	56/29^a^	10/8^a^	66/37^a^
3	Yu	2015	China	Asia	346	64.5 ± 9.2	Gastric cancer	Fluorouracil (5FU) and folinic acid chemotherapy	NR	WHO	ORR	141/107^a^	_	_	66/32^a^	88/79^a^	_	_	119/60^a^
4	Zhong	2015	China	Asia	263	62.40 ± 9.50	Gastric cancer	FOLFOX chemotherapy	I-IV	WHO	ORR	_	_	_	_	86/57^a^	57/45^a^	9/9^a^	66/54^a^
5	Ding	2015	China	Asia	380	58.7 ± 16.3	Gastric cancer	Platinum-based chemotherapy	I-IV	WHO	ORR	92/67^a^	_	_	131/90^a^	86/79^a^	_	_	137/78^a^
7	Xue	2015	China	Asia	410	63.7 ± 11.4	Gastric cancer	FOLFOX chemotherapy	I-IV	WHO	ORR	89/69^a^	123/52^a^	57/20^a^	_	108/66^a^	123/65^a^	37/11^a^	_
8	Yu	2015	China	Asia	228	55.7 ± 13.8	Gastric cancer	FOLFOX chemotherapy	I-IV	WHO	ORR	86/50^a^	46/32^a^	7/7^a^	_	79/42^a^	54/38^a^	6/9^a^	_
9	Zhou	2014	China	Asia	415	56.2 ± 15.6	Gastric cancer	Platinum-based chemotherapy	I-IV	WHO	ORR	139/109^a^	74/52^a^	25/16^a^	_	130/119^a^	67/46^a^	41/12^a^	_
11	Goekkurt	2009	Germany	Caucasian	156	64 (27–86)	Gastric cancer	FU and platinum-based	NR	WHO	ORR	66/91^a^	60/90^a^	36/88^a^	_	70/82^a^	48/123^a^	60/60^a^	_
12	Keam	2008	South Korea	Asia	73	59 (24–77)	Gastric cancer	FOLFOX chemotherapy	NR	WHO	ORR	28/4^a^	_	_	34/7^a^	1/7^a^	31/34^a^	_	_
13	Ruzzo	2006	Italy	Caucasian	175	61 (38–79)	Gastric cancer	Fluorouracil/cisplatin palliative chemotherapy	I-IV	RECIST	ORR	17/37^a^	36/54^a^	17/14^a^	_	13/34^a^	36/52^a^	21/19^a^	_

NR: not reported; ORR: objective response rate; OS: overall survival; RECIST: Response Evaluation Criteria in Solid Tumors; WHO: World Health Organization; ^a^number of patients for ORR; in front of oblique line is responders (complete response (CR) + partial response (PR)) and behind oblique line is nonresponder (stable disease (SD) + progressive disease (PD)).

**Table 3:** Meta-analysis of the association between ERCC2 rs13181 polymorphism and chemotherapy in objective response rate and overall survival for gastric cancer patients.

Genetic comparisons	Subgroup analysis	No. of studies	Test of association			Model	Test of heterogeneity		*P*_Egger_
			OR/HR (95% CI)	*Z*	*P*		*P*	*I* ^2^ (%)	
Objective response rate (OR)									
CC vs. AA	Total ethnicity	7	1.01 (0.61–1.68)	0.04	0.968	R	0.003	69.30	0.892
	Caucasian	2	1.16 (0.26–5.27)	0.20	0.845	R	0.004	88.20	
	Asian	5	1.00 (0.58–1.74)	0.00	0.997	R	0.047	58.50	
AC vs. AA	Total ethnicity	7	1.06 (0.83–1.37)	0.47	0.641	R	0.143	37.40	0.517
	Caucasian	2	1.06 (0.70–1.61)	0.28	0.783	R	0.290	10.70	
	Asian	5	1.04 (0.74–1.46)	0.25	0.806	R	0.077	52.60	
CC + AC vs. AA	Total ethnicity	10	1.07 (0.84–1.36)	0.52	0.600	R	0.017	55.40	0.661
	Caucasian	2	1.07 (0.48–2.39)	0.17	0.866	R	0.040	76.30	
	Asian	8	1.08 (0.83–1.41)	0.58	0.562	R	0.037	53.20	
CC vs. AA + AC	Total ethnicity	7	0.98 (0.65–1.46)	0.12	0.907	R	0.031	56.70	0.760
	Caucasian	2	1.06 (0.31–3.66)	0.09	0.925	R	0.006	86.70	
	Asian	5	1.02 (0.70–1.50)	0.12	0.907	F	0.270	22.60	

Overall survival (HR)									
CC vs. AA	Asian	5	0.99 (0.72–1.36)	0.09	0.928	F	0.185	35.4	0.739
AC vs. AA	Asian	5	1.01 (0.78–1.30)	0.07	0.947	F	0.468	0	0.651
CC + AC vs. AA	Asian	6	1.28 (0.85–1.91)	1.18	0.238	R	0.073	50.4	0.814

OR: odds ratio; HR: hazard ratio; CI: confidence interval; vs.: versus; F: fixed effect model; R: random effect model.

**Table 4:** Meta-analysis of the association between ERCC2 rs1799793 polymorphism and chemotherapy in objective response rate and overall survival for gastric cancer patients.

Genetic comparisons	Subgroup analysis	No. of studies	Test of association			Model	Test of heterogeneity		*P*_Egger_
			OR/HR (95% CI)	*Z*	*P*		*P*	*I* ^2^ (%)	
Objective response rate (ORR)									
AA vs. GG	Total ethnicity	8	1.17 (0.70–1.95)	0.59	0.556	R	0.002	69.2	0.348
	Caucasian	2	1.70 (0.71–4.05)	1.19	0.234	R	0.080	67.4	
	Asian	6	0.98 (0.48–1.98)	0.06	0.950	R	0.002	74.1	
GA vs. GG	Total ethnicity	9	0.94 (0.69–1.27)	0.41	0.679	R	0.012	59.1	0.259
	Caucasian	2	0.88 (0.23–3.38)	0.19	0.805	R	0.003	89.0	
	Asian	7	1.01 (0.82–1.24)	0.06	0.955	F	0.299	17.2	
AA + GA vs. GG	Total ethnicity	10	1.12 (0.85–1.46)	0.79	0.429	R	0.001	68.9	0.661
	Caucasian	2	1.15 (0.39–3.42)	0.26	0.797	R	0.009	85.5	
	Asian	8	1.13 (0.86–1.49)	0.87	0.384	R	0.006	64.8	
AA vs. GG + GA	Total ethnicity	8	1.24 (0.81–1.92)	0.98	0.325	R	0.009	62.6	0.053
	Caucasian	2	1.79 (1.24–2.57)	3.13	0.002	F	0.794	0	
	Asian	6	1.02 (0.54–1.90)	0.05	0.962	R	0.006	69.2	

Overall survival (HR)									
GA vs. GG	Asian	8	1.20 (0.96–1.51)	1.61	0.108	F	0.577	0	0.900
AA vs. GG	Asian	6	1.77 (1.20–2.60)	2.89	0.004	F	0.227	27.7	0.959
GA + AA vs. GG	Asian	4	1.62 (1.26–2.09)	3.76	<0.001	F	0.479	0	0.032

OR: odds ratio; HR: hazard ratio; CI: confidence interval; vs.: versus; F: fixed effect model; R: random effect model.

**Table 5:** Association between the ERCC2 rs13181 polymorphism and overall survival of chemotherapy in gastric cancer patients.

Study	Year	Country	Outcomes	ERCC2 rs13181				ERCC2 rs1799793			
				AA	AC	CC	AC + CC	GG	GA	AA	GA + AA
Zheng	2016	China	OS	1.0 (ref.)	1.10 (0.56–2.15)	1.37 (0.46–3.80)	—	1.0 (ref.)	1.12 (0.56–2.22)	1.38 (0.46–3.80)	—
Mo	2015	China	OS	1.0 (ref.)	1.14 (0.58–2.28)	1.35 (0.48–3.53)	1.20 (0.64–2.25)	1.0 (ref.)	1.12 (0.57–2.20)	1.93 (0.58–6.00)	1.25 (0.66–2.34)
Yu	2015	China	OS	1.0 (ref.)	—	—	1.57 (0.93–2.65)	1.0 (ref.)	—	—	1.78 (1.13–2.81)
Zhong	2015	China	OS	—	—	—	—	1.00 (ref)	1.15 (0.63–2.10)	1.76 (0.53–5.38)	1.23 (0.69–2.18)
Ding	2015	China	OS	1.0 (ref.)	—	—	1.05 (0.68–1.62)	1.0 (ref.)	—	—	1.97 (1.28–3.03)
Liu	2015	China	OS	1.0 (ref.)	—	—	1.12 (0.44–2.88)	1.0 (ref.)	2.12 (0.89–5.08)	—	—
Xue	2015	China	OS	1.0 (ref.)	0.66 (0.39–1.11)	0.44 (0.20–0.91)	—	1.0 (ref.)	0.82 (0.49–1.38)	0.75 (0.31–1.71)	—
Yu	2015	China	OS	1.0 (ref.)	1.31 (0.73–2.36)	1.75 (0.57–5.40)	—	1.0 (ref.)	1.52 (0.75–2.86)	3.86 (1.36–12.23)	—
Keam	2008	South Korea	OS	1.0 (ref.)	—	—	0.49 (0.17–1.37)	1.0 (ref.)	0.80 (0.28–2.26)	—	—

OS: overall survival.
